# Aleya cavernae gen. nov., sp. nov., and Lacrimispora cavernae sp. nov., novel taxa isolated from bat guano

**DOI:** 10.1099/ijsem.0.007260

**Published:** 2026-08-18

**Authors:** Samuel L. Miller, Nam Lu, Payton R. Shears, Jacobey King, Krithivasan Sankaranarayanan, Paul A. Lawson

**Affiliations:** 1 University of Oklahoma, School of Biological Sciences, 730 and 770 Van Vleet Oval, Norman, OK 73019, USA; 2 Laboratories of Molecular Anthropology and Microbiome Research, Stephenson Research and Technology Center, 101 David L. Boren Blvd, Norman, OK 73019, USA

**Keywords:** *Aleya cavernae*, bats, enrichment, guano, *Lacrimispora cavernae*, novel, taxonomy

## Abstract

Bats, of the order *Chiroptera*, comprise over 1,400 species and account for over 21% of all currently described mammalian species. However, despite this huge group of mammals, a remarkably small number of bacterial species have been described from this group. In a continuing study of non-human mammals, dozens of strains were isolated from anaerobic guano-pile enrichments collected in a bat cave near Protem, MO, USA. Of these, two novel taxa were identified and designated BS-2^T^ and BS-20^T^; both were rod-shaped, Gram-stain-positive bacteria. Phylogenetic analysis of the 16S rRNA gene and phylogenomic analysis placed BS-2^T^ within the genus *Lacrimispora*, and BS-20^T^ was located within the family *Jonesiaceae*, but with no particular affiliation to any genus. Overall genomic-based relatedness indices indicated that strains BS-2^T^ and BS-20^T^ shared average nucleotide identity and digital DNA–DNA hybridization values <95.0% and <70.0% with their nearest neighbouring taxa, which are the currently accepted thresholds for species-level delineation. Further, for strain BS-20^T^, genus-specific delineation indices demonstrated a shared average amino acid identity, and percentages of conserved proteins were <68.0% and <42.0%, respectively, between BS-20^T^ and other *Jonesiaceae* taxa, below commonly accepted genus-level delineation thresholds. The predominant cellular fatty acid of isolate BS-2^T^ was C_16:0_, whereas BS-20^T^ contained C_15:0_ anteiso and C_14:0_ as the predominant cellular fatty acids. The G+C content of BS-2^T^ and BS-20^T^ was 44.1 and 56.5 mol%, respectively. Based on biochemical, phylogenetic, genotypic and chemotaxonomic criteria, strains BS-2^T^ and BS-20^T^ represent novel isolates within the genus *Lacrimispora* and family *Jonesiaceae*, for which the names *Lacrimispora cavernae* sp. nov. and *Aleya cavernae* gen. nov., sp. nov. are proposed. Strains BS-2^T^ (=NRRL B 65694^T^=CCUG 77247^T^=CCM 9410^T^) and BS-20^T^ (=NRRL B-65699^T^=CCUG 77269^T^) are proposed as the type species of these novel taxa.

## Data availability

The GenBank/EMBL/DDBJ accession numbers for the 16S rRNA gene sequences of strains BS2^T^ and BS-20^T^ are PP809807.1 and PP337727.1. The GenBank/EMBL/DDBJ accession numbers for the genome sequences are GCA_040207125.1 and GCA_039995105.1, respectively.

## Introduction

With more than 1,400 recognized species, bats belong to the order *Chiroptera* and make up over 21% of all currently described mammals [1, 2]. However, despite these very large numbers, a remarkably small number of novel bacterial species have been described from this mammalian order; most of these organisms belong to the class *Actinomycetes* of the phylum *Actinomycetota* [3–8]. The reclassification of the class *Actinomyetia* (formerly *Actinomycetes*) within the phylum *Actinomycetota* occurred in 1997 [9]; within this restructuring, the family *Jonesiaceae* was created, which at the time of writing contains five genera with validly published names: *Jonesia* [10], *Sanguibacter* [11], *Falvimobilis* [12] and *Timonella* [13] according to the List of Prokaryotic Names with Standing in Nomenclature (https://lpsn.dsmz.de/family/Jonesiaceae, October 2025). Taxa belonging to this family have been isolated primarily from environmental sediments, blood samples and human faecal material [14]. Since 2020, *Lacrimispora* has had standing in the nomenclature [15] and currently comprises 11 species with validly published names (https://lpsn.dsmz.de/genus/Lacrimispora, October 2025), which are structurally Gram-stain-positive, obligately anaerobic, rod-shaped, spore-forming bacteria [15]. *Lacrimispora* have been primarily isolated from environmental samples, except for *Lacrimispora aerotolerans*, which was isolated from the rumen of a sheep [16]. Cultivated representatives from non-human mammalian microbiomes are underrepresented in the literature [17–19]. Recent studies have shown that understudied mammalian species, such as bats, have the potential to significantly increase our understanding of the diversity of the mammalian gut microbiome [1, 2]. In a continuing study of non-human mammalian microbiomes [20, 21] using a polyphasic taxonomic approach, this study describes two novel bacterial species, isolated from bat guano, within the genus *Lacrimispora* and the family *Jonesiaceae*. This work contributes to our understanding of the diversity and microbial ecology of the bat guano microbiome. Here, we report the first bacteria isolated from *Lacrimispora* or *Jonesiaceae* in bat guano; additionally, we also report the first obligately anaerobic bacterium (BS-2^T^) from bat guano. Strains BS-2^T^ and BS-20^T^ represent novel species within the genus *Lacrimispora* and the family *Jonesiaceae. Lacrimispora cavernae* sp. nov. and *Aleya cavernae* gen. nov., sp. nov. are herein proposed.

### Isolation and ecology

Strains BS-2^T^ and BS-20^T^ were isolated from a core sample obtained from a guano pile within Tumbling Creek Cave, a privately owned cave system in Protem, MO, USA (36.6° N 92.8° W). The core sample was aseptically collected, processed and placed in an anaerobic jar (Oxoid, Basingstoke, Hampshire, UK) with an anaerobic gas pack (BD Difco, Franklin Lakes, NJ, USA). The jar was placed on ice and transported to the Lawson Microbial Systematics Laboratory at the University of Oklahoma in Norman for further processing. Multiple anoxic enrichments using cellulose, xylan or the exoskeleton from a deceased house cricket (*Acheta domesticus*) as sole substrates. These enrichments were constructed and inoculated with 1.0 ml of a guano slurry (1.0 g of bat guano and 9 ml of 1× sterile PBS). Enrichments were carried out in a sealed serum bottle containing 100 ml modified Medium 2, which consisted of (per 100 ml distilled water) casitone (1.0 g), yeast extract (0.25 g), mineral solution (A) (15.0 ml), mineral solution (B) (15.0 ml), clarified rumen fluid (20 ml), resazurin (0.0001 g), sodium lactate (70% w/v) (1.0 g), cellulose, xylan or exoskeleton (0.2 g), cysteine HCl (0.05 g), sodium bicarbonate (0.4 g) and distilled water (to 1000 ml). Mineral solution (A) contained (per 1,000 ml) K_2_HPO_4_ (3.0 g). Mineral solution (B) contained (per 1000 ml) KH_2_PO_4_ (3.0 g), (NH_4_)_2_SO_4_ (6.0 g), NaCl (6.0 g), MgSO_4_·7H_2_O (0.6 g) and CaCl_2_ (0.6 g) as previously described [20, 21]. The enrichments were incubated at 4, 25 and 37 °C under anoxic conditions with a headspace of 5% hydrogen, 10% carbon dioxide and 85% nitrogen for 14 days. After incubation, samples were inoculated onto tryptic soy agar (TSA) (BD Difco) supplemented with 5% defibrinated sheep’s blood and subsequently subjected to multiple plate subcultures under anoxic conditions at 4, 25 and 37 °C until pure isolates were obtained as determined by Gram-stain reaction and phase contrast microscopy (CX41, Olympus, Center Valley, PA, USA) at 1,000× magnification. Two of the resulting isolates were designated BS-2^T^, which was isolated from a xylan enrichment at 37 °C, and BS-20^T^, which was isolated at 25 °C from an enrichment containing a house cricket (*A. domesticus*) exoskeleton.

While supplements were not required for growth, better growth and thus biomass yield were achieved by incorporating them into the media. For example, BS-2^T^ could grow on Reinforced Clostridial Medium agar (BD Difco) and Yeast Casitone Fatty Acids Broth with Carbohydrates (Anaerobe Systems, Morgan Hill, CA, USA) without blood or rumen fluid as supplements. For BS-20^T^ using TSA/tryptic soy broth (TSB) (BD Difco), rumen fluid is not required for growth. After primary isolation, BS-2^T^ was maintained under anoxic conditions on Brain Heart Infusion (BHI) plus 5 % sheep’s blood medium at 37 °C and preserved in BHI plus 5% clarified rumen fluid with 20 % (v/v) glycerol, whereas BS-20^T^ was maintained under aerobic conditions on TSA plus 5% sheep’s blood medium between 25 °C and preserved in TSB with 20 % (v/v) glycerol. Both isolates were stored following snap freezing at −80 °C.

### 16S rRNA gene sequencing and phylogenetics

**Fig. 1.**
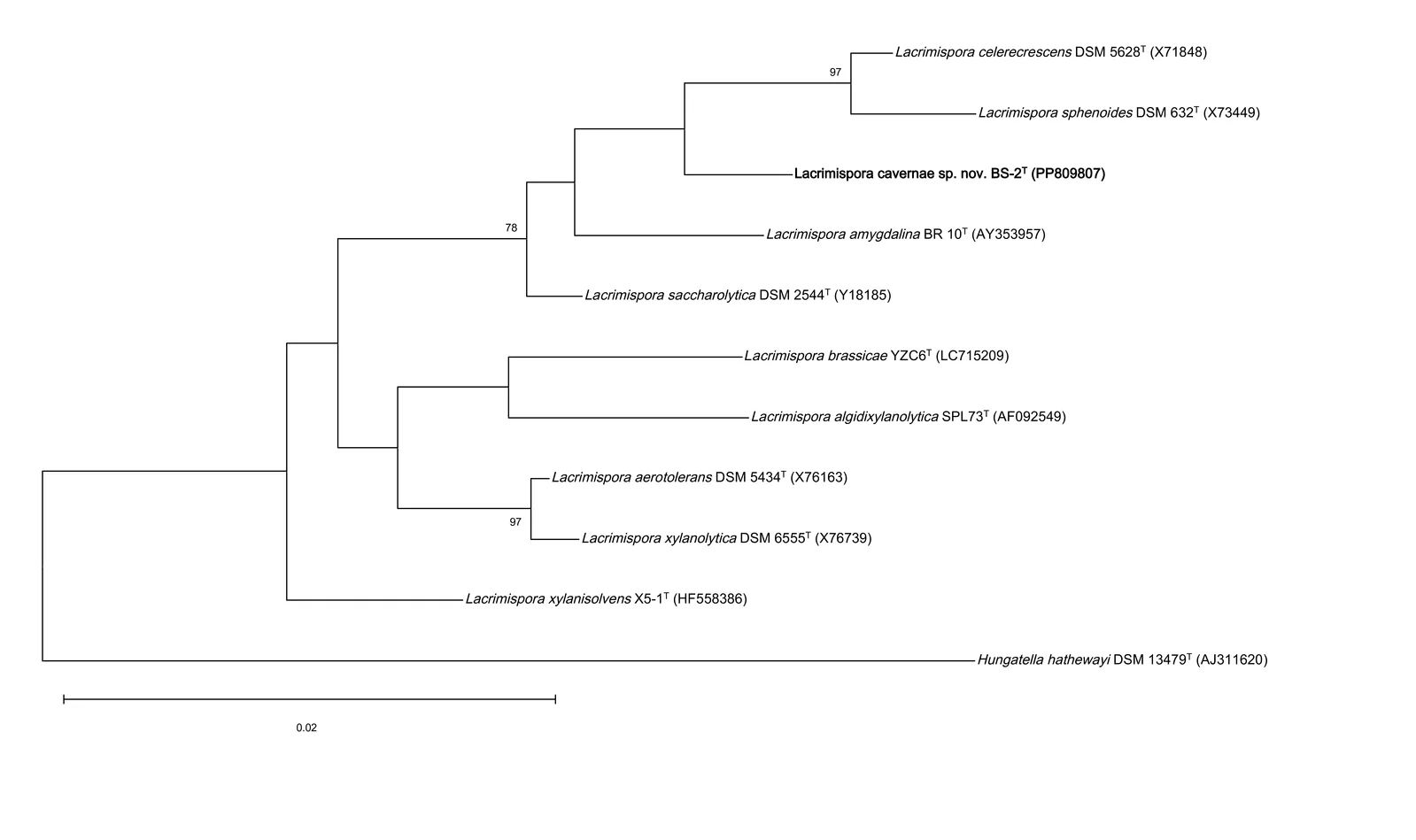
Maximum-likelihood phylogenetic tree based on 16S rRNA gene sequences showing the relationship of BS-2^T^ within *Lacrimispora*. Bootstrap values (%) were obtained with 1,000 replicates and are displayed at their branch nodes. *Hungatella hathewayi* DSM 13479^T^ (AJ311620) was used as an outgroup. Bar indicates 0.02 substitutions per site.

**Fig. 2.**
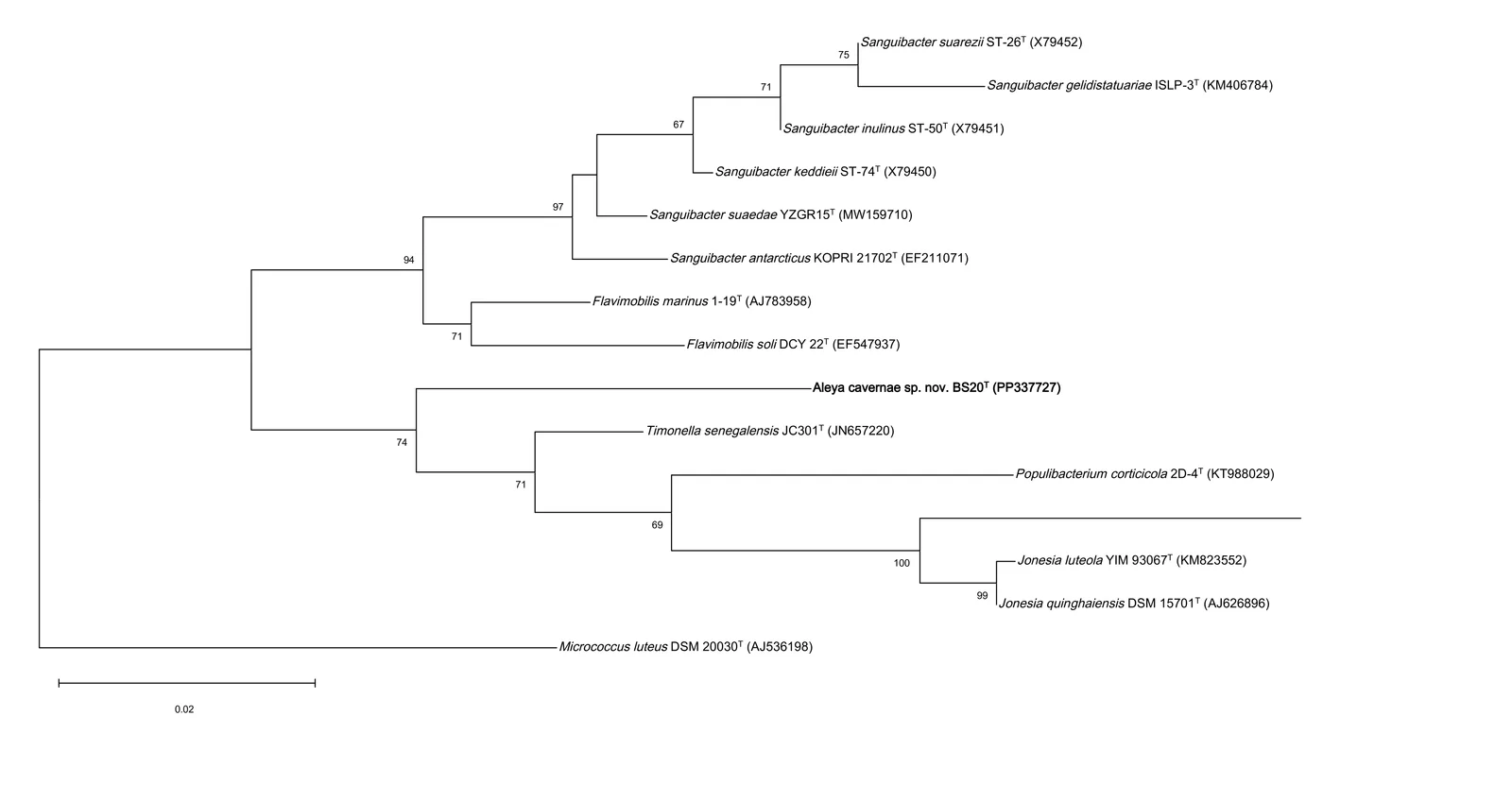
Maximum-likelihood phylogenetic tree based on 16S rRNA gene sequences showing the relationships between strain BS-20^T^ within *Jonesiaceae*. Bootstrap values (%) were obtained with 1,000 replicates and are displayed at their branch nodes. *Micrococcus luteus* NCTC 2665^T^ (AJ536198) was used as the outgroup. Bar indicates 0.02 substitutions per site.

Strains BS-2^T^ and BS-20^T^ were sent to GENEWIZ (South Plainfield, NJ, USA), where near full-length 16S rRNA gene sequences were obtained using Sanger sequencing [22]. The EzBioCloud platform (https://www.ezbiocloud.net/) was used to compare the 16S rRNA gene sequences of BS-2^T^ and BS-20^T^ with those of the most closely related type strains, as determined by a blast search against the GenBank database [23–25]. Downloaded sequences were aligned using muscle [26], and a 16S rRNA phylogenetic tree was reconstructed using mega 12 [27] using the maximum-likelihood method [28] and the Tamura–Nei substitution model [29] with 1,000 bootstrap replications. The phylogenetic analysis demonstrated that strain BS-2^T^ was closely related to *Lacrimispora saccharolytica* WM1^T^ (98.6%) (Fig. 1) and shared 16S rRNA gene similarity values<98.7% with its close relatives (Table S1, available in the online Supplementary Material). For strain BS-20^T^, phylogenetic analyses indicated that BS-20^T^ was equidistantly distinct from other members within the family *Jonesiaceae* (Fig. 2) but shared the closest 16S rRNA gene sequence similarity with *Timonella senegalensis* JC301^T^ (94.8%), *Sanguibacter keddieii* ST-74^T^ (93.3%) and *Flavimobilis marinus* JCM 12547^T^ (93.3%) (Table S2).

### Whole-genome sequencing and genomic analyses

**Fig. 3.**
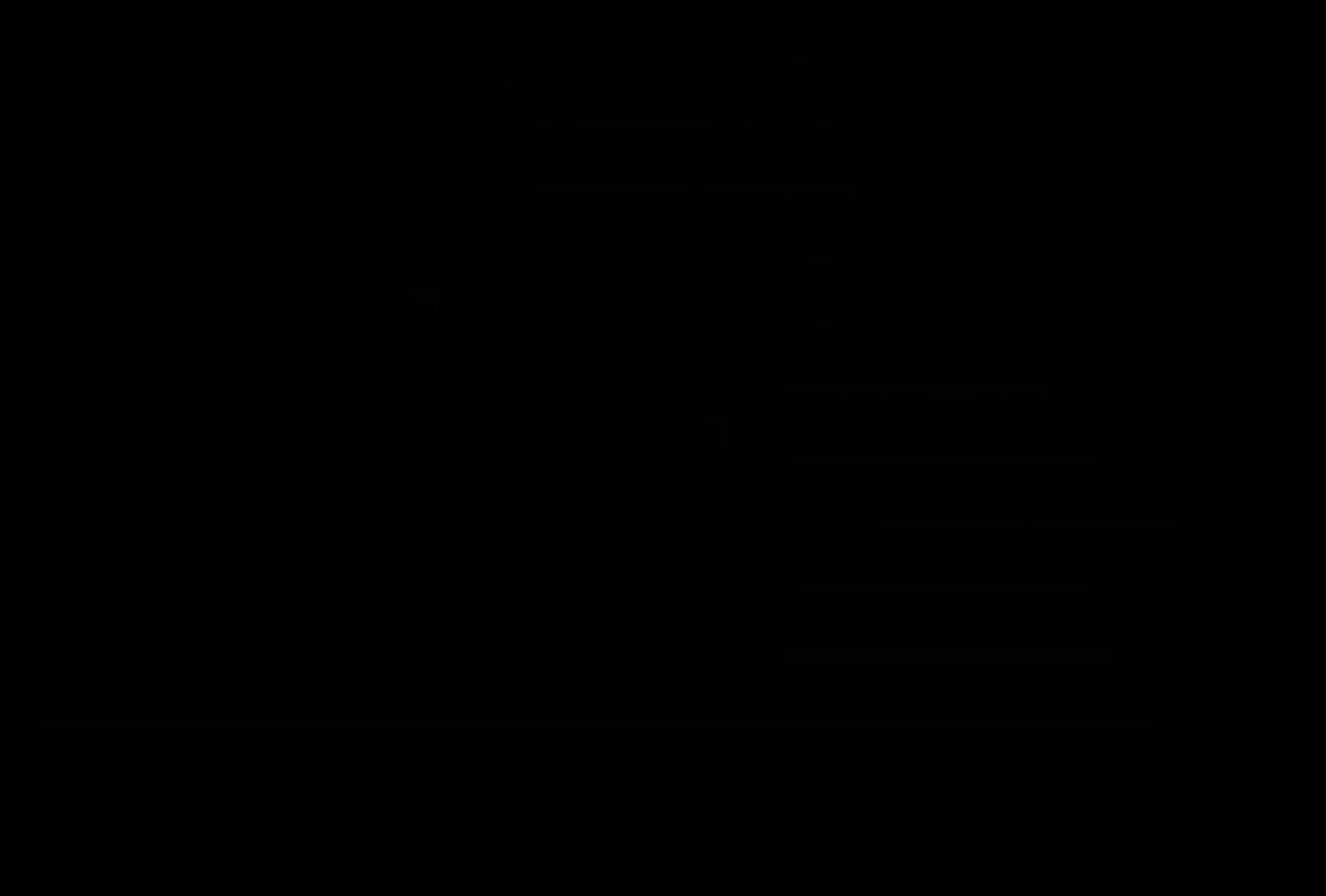
Maximum-likelihood phylogenetic tree based on 844 single-copy genes showing the relationships between BS-2^T^ within *Lacrimispora*. A total of 812,343 aligned nucleotides and 270,781 amino acids were used to build this tree. Bootstraps were generated with 100 rounds of the ‘Rapid’ bootstrapping option within RAxML. *Hungatella hathewayi* DSM 13479^T^ (GCA_025149285.1) was used as an outgroup. Bar indicates the mean number of substitutions per site, 0.2.

**Fig. 4.**
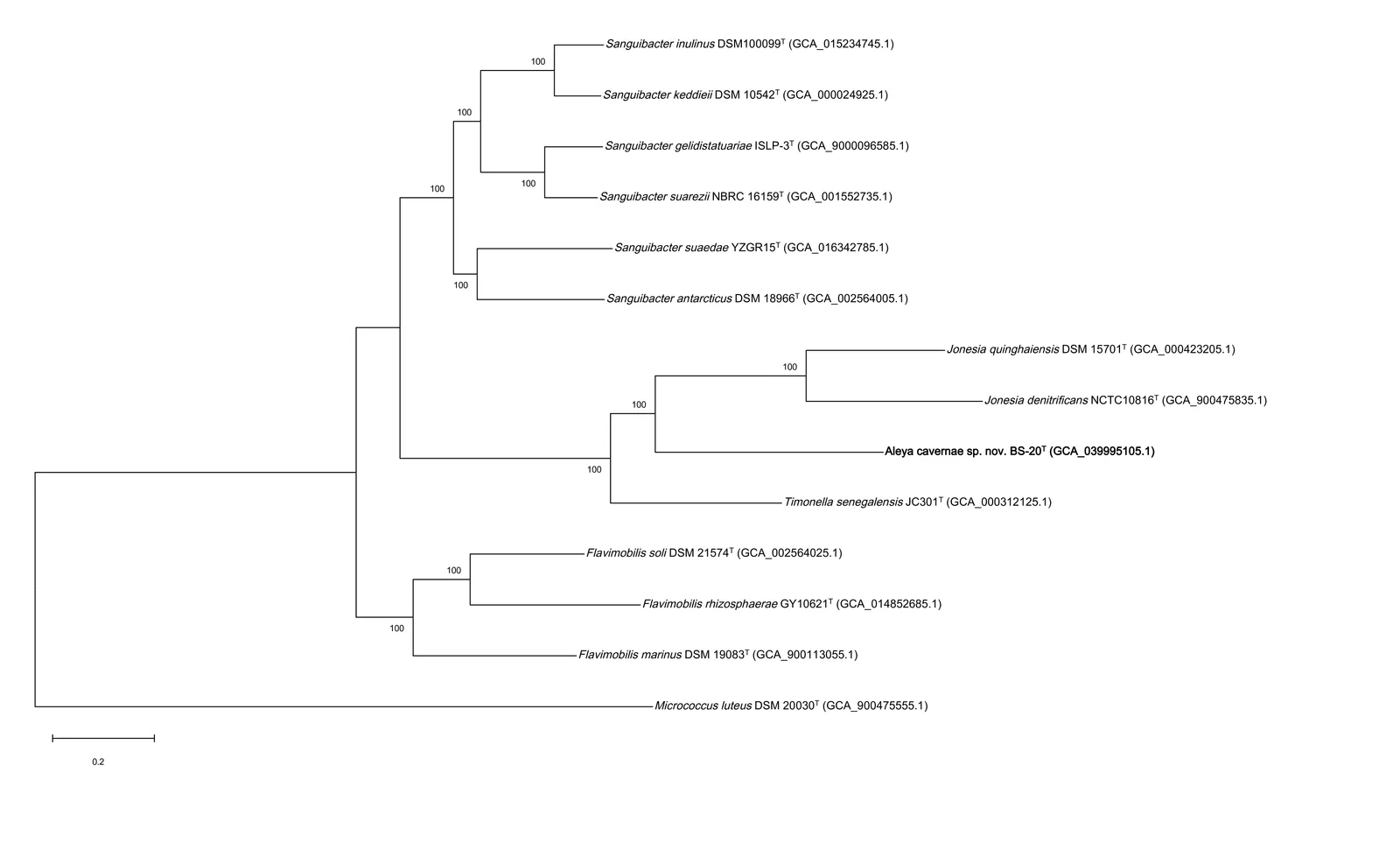
Maximum-likelihood phylogenetic tree based on 479 single-copy genes, showing the relationships between strain BS-20^T^ within *Jonesiaceae*. A total of 575,724 aligned nucleotides and 191,894 amino acids were used to build this tree. Bootstraps were generated with 100 rounds of the ‘Rapid’ bootstrapping option within RAxML. *Micrococcus luteus* DSM20030^T^ (GCA_019890915.1) was used as the outgroup. Bar indicates the mean number of substitutions per site, 0.2.

Cell biomass for BS-2^T^ and BS-20^T^ was harvested from an overnight culture, and the cell biomass was stored in DNA/RNA Shield (Zymo, Irvine, CA, USA). DNA extraction, sequencing and annotation were conducted using the services of a commercial provider (Plasmidsaurus, Eugene, OR, USA). gDNA was extracted with the Quick-DNA Fungal/Bacterial Miniprep Kit (Zymo, Irvine, CA, USA). Oxford Nanopore Technologies long-read libraries were prepared using amplification-free methods with v14 chemistry, then sequenced on an R10.4.1 flow cell. Default parameters were used for all software unless otherwise specified. Reads were filtered using Filtlong v0.2.1 (https://github.com/rrwick/Filtlong), with a minimum Phred score of 15 and a minimum length of 1,000 bp. The genome was assembled and polished using Flye v2.9.1 [30] and Medaka v1.8.0 (https://github.com/nanoporetech/medaka), respectively. Assembly completeness and contamination were assessed using CheckM v1.2.2 [31], and genome annotation was performed using the NCBI Prokaryotic Genome Annotation Pipeline (PGAP) v6.7 [32]. Examination the 16S rRNA gene sequences derived from WGS and Sanger sequencing method, demonstrated that in the sequences obtainedfor strain BS-2T, there was 1one instance where an A was called in the Sanger method and a G was called in the WGS-derived 16S rRNA gene sequence; otherwise, Sanger-derived and WGS-derived 16S rRNA gene sequences for BS-2T and BS-20T were identical. Phylogenomic tree analyses of strains BS-2^T^ and BS-20^T^ were performed in a custom workspace on the Bacterial and Viral Bioinformatics Resource Center [33] (BV-BRC) (https://www.bv-brc.org/). The phylogenomic trees for BS-2^T^ (Fig. 3) and BS-20^T^ (Fig. 4) were constructed using the CodonTree [34] method within BV-BRC [33] and the rapid RAxML analysis [35] v8.2.11. Support values for the phylogenomic trees were generated using 100 rounds of the ‘Rapid bootstrapping’ option in RAxML, and the trees were visualized with Interactive Tree Of Life v6 [34]. To delineate species boundaries for BS-2^T^ and BS-20^T^, overall genomic relatedness indices (OGRIs) between the isolates and their close relatives were assessed according to the most recent proposed minimal standards for the use of genome data in prokaryotic taxonomy [35]. Average nucleotide identity (ANI) [36, 37] and digital DNA–DNA hybridization (dDDH) values [38, 39] were computed using EzBioCloud’s ANI Calculator and the Genome-to-Genome Distance Calculator v3.0 [25, 37]. Genomic data were also used to determine the relationship between BS-20^T^ and all other members of the family *Jonesiaceae*. The genus-specific delineation indices, average amino acid identity (AAI) and percentage of conserved proteins (POCP) were calculated using the AAI calculator [40] and a custom shell script based on previously defined parameters [41]. Once assembled, the draft genome sequences of BS-2^T^ and BS-20^T^ consisted of 1 contig. Genome lengths of 4.5 and 3.5 Mbp were observed for BS-2^T^ and BS-20^T^, respectively; the G+C content was 44.1 mol% and 56.5 mol%, respectively. Of the 4174 predicted genes for BS-2^T^, 4038 were protein-coding sequences (CDSs), 18 rRNA genes, 69 tRNAs, 1 ncRNA and 45 pseudogenes were identified. For BS-20^T^, 3165 genes were predicted, of which 3063 were CDSs, 16 rRNA genes, 56 tRNAs and 27 pseudogenes were identified. The phylogenomic relationships between BS-2^T^ and its relatives (Fig. 3) revealed that BS-2^T^ was nested within *Lacrimispora*, clustering most closely with *Lacrimispora saccharolytica* WM1^T^ (GCA_000144625.1). For BS-20^T^, phylogenomic analysis (Fig. 4) demonstrated its taxonomical distinctness from members of the family *Jonesiaceae*. Strains BS-2^T^ and BS-20^T^ shared ANI and dDDH values of <95.0% and <70.0% with their close relatives (Tables S1 and S2). Further intergenic comparisons for BS-20^T^ revealed AAI values between 61.7 and 67.8%, whereas POCP values were between 35.9 and 41.9% for BS-20^T^ and closely related, representative relatives of different genera within *Jonesiaceae* (Table S2). The main genotypic differential characteristics of BS-2^T^ and BS-20^T^ and their close relatives are given in Tables S1 and S2, respectively.

### Physiological and chemotaxonomic characterization

The Gram-stain reaction was determined using the Advanced Gram Stain Kit (Hardy Diagnostics, Santa Maria, CA, USA) following the manufacturer’s instructions. Aerotolerance was determined using thioglycolate medium (BD Difco). Cells were examined for motility and endospore production using phase contrast microscopy (CX41, Olympus, Center Valley) at 1,000 x magnification. Temperature ranges for growth were determined in TSB or BHI supplemented with 5% (v/v) clarified rumen fluid at 4, 20, 25, 30, 37, 45, 50 and 55 °C. Salt tolerance was determined in TSB or BHI broth supplemented with 5% (v/v) clarified rumen fluid and tested at 0.5% (w/v) NaCl and between 1.0% (w/v) and 10.0% (w/v) in increments of 1.0% (w/v); pH ranges for growth were determined in TSB or BHI broth supplemented with 5% (v/v) clarified rumen fluid between 5.0 and 9.0, in increments of 1.0, and all tests were performed in triplicate. For the pH ranges, the following buffers were prepared at a final concentration of 10 mM: sodium acetate (pH 5.0), potassium phosphate (pH 6.0–8.0) and Tris-HCl (pH 9.0). Optimal growth conditions were determined by OD at 600 nm using a spectrophotometer (Spectronic 20D+, Milton Roy, Houston, TX, USA) or a BioTek 800 TS Absorbance Reader (Agilent, Santa Clara, CA, USA). An increase in OD greater than 0.1 after 5 days of incubation was considered growth. Additional biochemical traits were determined using the API rapid ID32A (BS-2^T^) and API-ZYM (BS-20^T^) test systems (bioMérieux, Durham, NC, USA), according to the manufacturer’s instructions. Chemotaxonomic features were determined at the Centre for Microbial Identification and Taxonomy (University of Oklahoma, Norman, Oklahoma, USA). For cellular fatty acids analysis, strain BS-2^T^ was grown anaerobically on BHI plus 5% sheep’s blood medium at 37 °C for 2 days, whereas BS-20^T^ was grown aerobically on TSA plus 5% sheep’s blood medium at 30 °C for 2 days. Biomass was harvested, extracted and analysed using the Microbial Identification System (MIDI) [42, 43]. Analysis was performed with an Agilent Technologies 6890 N gas chromatograph equipped with a phenyl methyl silicone fused silica capillary column (HP-Ultra 2.25 m×0.2 mm×0.33 µm film thickness) coupled with a flame ionization detector. Hydrogen was used as the carrier gas. The temperature programme was preset at 170 °C and increased at 5 °C min^−1^ to a final temperature of 270 °C. Fatty acids were identified and expressed in percentages using the QBA1 peak naming database within the MIDI system.

The cells of BS-2^T^ are Gram-stain-positive, motile, obligately anaerobic rods. Colonies on BHI with 5% sheep’s blood medium were circular (2–3 mm) and grey on the agar surface after 48 h at 37 °C. Cells produced endospores, which were visible under phase-contrast microscopy. Strain BS-2^T^ grows between 20 and 45 °C, with optimal growth at 37 °C. Additionally, the strain can grow at NaCl concentrations up to 3.0% (w/v), with optimal growth at 0.5% (w/v). It also grew between pH 6.0 and 8.0, with an optimum at pH 7.0. The API rapid ID32A test system detected positive reactions for indole, nitrate reduction, raffinose, mannose, *N*-acetyl-*β*-glucosaminidase, *β*-glucuronidase, *α*-arabinosidase, *β*-glucosidase, *α*-glucosidase, *β*-galactosidase and *β*-galactosidase-phosphatase. Negative reactions were detected for serine arylamidase, glutamyl glutamic acid arylamidase, histidine arylamidase, *α*-fucosidase, glutamic acid decarboxylase, glycine arylamidase, alanine arylamidase, tyrosine arylamidase, pyroglutamic acid arylamidase, leucine arylamidase, phenylalanine arylamidase, leucyl glycine arylamidase, proline arylamidase, arginine arylamidase, alkaline phosphatase, indole, nitrate reduction, raffinose, mannose, *N*-acetyl-*β*-glucosaminidase, *β*-glucuronidase, *α*-arabinosidase, *β*-glucosidase, *α*-glucosidase, *β*-6-gal-phosphatase, *β*-galactosidase, *α*-galactosidase, arginine dihydrolase and urease. The differential morphological and physiological characteristics of BS-2^T^ and its close relatives are detailed in Table 1. The only major fatty acid for strain BS-2^T^ was C_16 : 0_ (42.4%), which is consistent with other representative species of *Lacrimispora* (Table S3).

**Table 1.** Differential physiological characteristics of strain BS-2^T^ and close relatives Strains: 1, BS-2^T^; 2 (data from this study), *Lacrimispora saccharolytica* WM1^T^ [59]; 3, *Lacrimispora celerecrescens* DSM 5628^T^ [60]; 4, *Lacrimispora indolis* DSM 755^T^ [56]; 5, *Lacrimispora sphenoides* JCM 1415^T^ [61]. nr, not reported. The API rapid ID32A data for *Lacrimispora sphenoides* JCM 1415^T^ was taken from https://bacdive.dsmz.de/strain/2704. The following characteristics were shared between all isolates where data were reported: endospore production (+), serine arylamidase (−), glutamyl glutamic acid arylamidase (−), histidine arylamidase (−), *α*-fucosidase (−), glutamic acid decarboxylase (−), glycine arylamidase (−), alanine arylamidase (−), tyrosine arylamidase (−), leucine arylamidase (−), phenylalanine arylamidase (−), leucyl glycine arylamidase (−), proline arylamidase (−), alkaline phosphatase (−), nitrate reduction (−), *β*-glucuronidase (−), β-galactosidase 6-phosphate (−), arginine dihydrolase (−) and urease (−).

Characteristic	1	2	3	4	5
**Gram-stain reaction**	+	−	+	+	+
**Motility**	+	−	+	+	+
**Temperature range**	20–45	17–43	20–40	nr	nr
**Temperature optimum**	37	37	35	37	37
**pH range**	6.0–8.0	6.0–8.8	5.0–7.5	nr	nr
**pH optimum**	7.0	7.4	7.0	5.5	nr
**Salt range %**	0.5–3.0	nr	nr	nr	nr
**Salt optimum %**	0.5	nr	nr	nr	nr
**Isolation source**	Bat guano	Sewage sludge	Methanogenic enrichment	Soil	Human gangrene
**API rapid ID32A**					
**Pyroglutamic acid arylamidase**	−	nr	−	nr	+
**Arginine arylamidase**	−	nr	+/−	nr	+
**Indole**	+	nr	+/−	nr	−
**Raffinose**	+	nr	−	nr	−
**Mannose**	+	nr	−	nr	−
***N*-Acetyl-*β-*glucosaminidase**	+	nr	+/−	nr	−
***α*-Arabinosidase**	−	nr	+/−	nr	−
***β*-Glucosidase**	+	nr	+/−	nr	−
***α*-Glucosidase**	+	nr	+/−	nr	−
***β*-Galactosidase**	+	nr	+	nr	−
***α*-Galactosidase**	−	nr	+/−	nr	−

The cells of BS-20^T^ are Gram-stain-positive rods, motile and facultatively anaerobic. Colonies on TSA with 5% sheep’s blood medium were tiny (<1 mm), circular and white on the agar surface after 48 h at 25 °C. Endospores were not observed with phase-contrast microscopy. Strain BS-20^T^ can grow between 4 and 30 °C, with optimal growth at 25 °C. Additionally, the strain grew at NaCl concentrations ranging from 0.5% (w/v) to 4.0% (w/v), with optimal growth at 0.5% (w/v). It grew between pH 6.0 and 8.0, with an optimum at pH 7.0. The API ZYM test system detected positive reactions for esterase (C4), esterase lipase (C8), N-acetyl-*β*-glucosaminidase, *α*-mannosidase and naphthol-AS-BI-phosphohydrolase. Negative reactions were detected for alkaline phosphatase, acid phosphatase, lipase (C14), leucine arylamidase, valine arylamidase, cystine arylamidase, trypsin, *α*-chymotrypsin, *α*-galactosidase, *β*-galactosidase, *β*-glucuronidase, *α*-glucosidase, *β*-glucosidase and *α*-fucosidase. Differences were observed between BS-20^T^ and members of other genera in that BS-20^T^ did not possess valine arylamidase or *β*-glucosidase. The main morphological and physiological characteristics of strain BS-20^T^ and close relatives are given in Table 2. For strain BS-20^T^, anteiso-C_15 : 0_ (50.2%) and C_14:0_ (11.5%) were the major fatty acids detected (Table S4).

**Table 2.** Differential physiological characteristics of strain BS-20^T^ and close relatives Strains: 1, BS-20^T^ (data from this study); 2, *T. senegalensis* JC301T [13]; 3 *S. keddieii* ST-74^T^ [53, 62]; 4, *Jonesia denitrificans* DSM 20603^T^ [10, 63]; 5, *Populibacterium corticicola* KCTC 33576^T^ [52]; 6, *F. marinus* JCM 12547^T^ [62, 64]. nr, not reported. The following characteristics were shared between all isolates, where data were reported: Gram-stain reaction (+), endospore production (−), motility (+), lipase (C 14) (−), trypsin (−), *β*-glucuronidase (−), *N*-acetyl-*β*-glucosaminidase (+) and *α*-fucosidase (−). The API ZYM data for *S. keddieii* ST-74^T^, *Jonesia denitrificans* DSM 20603^T^ and *F. marinus* JCM 12547^T^ were taken from https://catalogue-crbip.pasteur.fr.

Characteristic	1	2	3	4	5	6
**Temperature range**	4–30 °C	30–37 °C	10–37 °C	10–40 °C	10–37 °C	15–37
**Temperature optimum**	25 °C	37 °C	25–30 °C	30–35 °C	28–30 °C	25–30
**pH range**	6.0–8.0	nr	7.2	7.0–9.0	6.0–10.0	5.5–9.0
**pH optimum**	7.0	nr	7.2	7.0–8.0	7.0–8.0	nr
**Salt range % (w/v**)	0.0–4.0	nr	0.0–0.5	0.0–9.0	0.0–3.0	0.0–7.0
**Salt optimum % (w/v**)	0.5	nr	0.0–0.5	0.0–2.0	nr	3.0
**Source**	Bat guano	Human faeces	Bovine blood	Boiled ox blood	Bark tissue	Costal sediment
**API ZYM**						
**Alkaline phosphatase**	−	nr	−	−	+	−
**Esterase (C 4**)	+	nr	+	−	+	+
**Esterase Lipase (C 8**)	+	nr	+	−	−	+
**Leucine arylamidase**	−	nr	+	−	+	+
**Valine arylamidase**	−	nr	+	+	+	+
**Cystine arylamidase**	−	nr	−	−	−	+
***α*-Chymotrypsin**	−	nr	+	−	−	+
**Acid phosphatase**	−	nr	+	−	+	+
**Naphthol-AS-BI-phosphatase**	+	nr	+	−	+	−
***α*-Galactosidase**	−	nr	+	−	+	−
*β* **-Galactosidase**	−	nr	+	−	+	+
***α*-Glucosidase**	−	nr	+	−	+	+
*β* **-Glucosidase**	−	nr	+	+	+	+
***α*-Mannosidase**	+	nr	+	−	−	−

revision.

Representative members of *Jonesiaceae*, such as *S. keddieii*, *Jonesia denitrificans*, *Populibacterium corticicola* and *F. marinus*, are consistent in also producing anteiso-C_15   0_ as a major product, with C_16:0_ also being a major product, although in BS-20^T^, this fatty acid is only present in moderate amounts. However, strain BS-20^T^, unlike the aforementioned species, can be distinguished by the presence of C_14:0_ as a major product. The main morphological and physiological characteristics of strain BS-20^T^ and close relatives are given in Table 2.

### *In silico* physiological and chemotaxonomic characterization

In this study, an *in silico* approach was used to investigate sporulation, cell wall structure and glycerophospholipid metabolism. Sporulation was determined by screening for the presence of the *spo0A* gene (EC 2.7.13.3), a master regulator of endospore formation [44], by using blast [23]. Additionally, by using blast [23], the diagnostic diamino acid encoding for either UDP-*N*-acetylmuramoylalanyl-d-glutamate-2,6-diaminopimelate ligase (EC 6.3.2.13) or UDP-*N*-acetylmuramoyl-l-alanyl-d-glutamate-l-lysine ligase (EC 6.3.2.7) was investigated. Polar lipid production was examined using the Kyoto Encyclopedia of Genes and Genomes (KEGG) database [45] and KEGG Mapper [46] as previously described [21]. *In silico* analysis is increasingly being used to derive phenotypic [47] and chemotaxonomic [21] traits from genome data [48] and is now routinely being used for species descriptions [49, 50], even at the genus level.

For BS-2^T^, the presence of the gene *spo0A* was detected, whereas for BS-20^T^, it was not. Regarding the diagnostic diamino acid, meso-diaminopimelic acid (EC 6.3.2.13) was detected for BS-2^T^, which is consistent with other members of the genus *Lacrimispora*, whereas l-lysine (EC 6.3.2.7) was detected for BS-20^T^, which is again consistent with members of the closely related genus *Jonesia*. No information is available for the chemotaxonomic properties of *Timonella senegalensis*, the sole member of the genus [13]. For polar lipids, BS-2^T^, the results (Fig. S1) indicate that, for phosphatidylglycerol (PG), of the two required enzymes (phosphatidylglycerophosphate synthase (EC 2.7.8.5) and phosphatidylglycerophosphate phosphatase (EC 3.1.3.27), the latter is missing. However, we have shown in earlier studies that this discrepancy between genomic data and results obtained in the laboratory is a common phenomenon with this enzyme missing in many genomes, leading to the hypothesis that an alternative enzyme (as yet unidentified) may replace Phosphatidylglycerophatase A [49, 51]. The predicted results for BS-2^T^ are PG and diphosphatidylglycerol (DPG). For BS-20^T^, the results (Fig. S1) indicate the genome contains the enzymes phosphatidylglycerophate synthetase (E.C. 2.7.8.5) and phosphatidylglycerophate phosphatase (E.C. 3.1.3.27) required to synthesize PG from the essential building block cytidine diphosphate diacylglycerol. Additionally, the enzyme cardiolipin synthase (E.C. 2.7.8.41) is present; this enzyme is required to produce DPG (cardiolipin). Therefore, on the basis of the results of this analysis, the major polar lipids predicted to be produced by BS-20^T^ are PG and DPG, consistent with those of other type species of genera within *Jonesiaceae* [10, 52, 53].

### Global ecological distribution

Finally, the ecological distribution patterns of BS-2^T^ and BS-20^T^ were assessed using Sandpiper [54] (v1.0.1), an interface that uses the SingleM tool to accurately map metagenomic reads to genomes. At the time of analysis, 707,499 distinct metagenomic datasets (from 32,370 projects) were analysed and made available through Sandpiper, with taxonomy annotations matching the Genome Taxonomy Database (GTDB) [54] release 226. Results were downloaded and used to assess the global distribution patterns (occurrence and relative abundance) and habitat preferences of BS-2^T^ and BS-20^T^. For assessing the distribution of BS-2^T^ and BS-20^T^, whole-genome sequences GCA_040207125.1 and GCA_039995105.1 were used, respectively. Our results identified BS-2^T^ and BS-20^T^ in 15 and 432 datasets, respectively (<0.1% of the total datasets analysed in Sandpiper). The percentage abundance of BS-2^T^ and BS-20^T^ did not exceed 3.5%. The largest number of datasets for BS-2^T^ came from engineered environments (40.0% of total), followed by aquatic environments (33.3%) and termite metagenomes (13.3%). The dataset with the highest abundance of BS-2^T^ (0.1%) was derived from a study [55] on the termite gut (PRJNA732531), whereas the largest number of datasets for BS-20^T^ came from host-associated environments (55.2% of total), followed by engineered (28.4%) and soil (12.3%) environments. The dataset with the highest abundance of BS-20^T^ (3.5%) was derived from a soil metagenome (PRJNA1026177).

### Taxonomic conclusions

In addition to the phylogenetic and phylogenomic reconstructions, strain BS-2^T^ produces C_16:0_ as the only major fatty acid (Table S3), which is consistent with other *Lacrimispora* spp. [56] and supports its placement within this genus. Strain BS-2^T^ shared 16S rRNA gene similarity values <98.7% with its close relatives, and phylogenetic analyses indicated that BS-2^T^ was distinct from other members within the *Lacrimispora* genus. The 16S rRNA gene sequence similarity values (Table S1) and phylogenetic tree (Fig. 1) support the proposal that strain BS-2^T^ represents a novel *Lacrimispora* species based on the routinely used 16S rRNA accepted threshold to demarcate this rank [57, 58]. Furthermore, in addition to the 16S rRNA analysis, OGRIs support the recognition of strain BS-2^T^ as a novel species, for example, ANI values with its closest relatives *Lacrimispora celerecrescens* DSM 5628^T^ and *Lacrimispora sphenoides* JCM 1415^T^ of 88.4 and 88.5% respectively, and dDDH values of 36.5% and 36.6% respectively are well below the cutoff values used for the differentiation of species [36, 38, 57].

Strain BS-20^T^ differed uniquely from other representative members of the family *Jonesiaceae* in that it could grow at 4 °C but could not grow at temperatures>30 °C (Table 2). Additionally, BS-20^T^ had a slightly lower G+C (mol%) content than other representative members (56.5 mol%). Some differences in enzymatic capability were also observed, with BS-20^T^ lacking valine arylamidase and *β*-glucosidase (Table 2). For strain BS-20^T^, anteiso-C_15 : 0_ was identified as a major fatty acid, which is consistent with other genera within the family *Jonesiaceae* [10–12]. However, differences were observed between BS-20^T^ and representative members of other *Jonesiaceae* genera in that BS-20^T^ also had C_14:0_ as a major fatty acid and did not have C_16:0_ as a major fatty acid (Table S4); these features were unique to BS-20^T^. For strain BS-20^T^, 16S rRNA analyses indicated that BS-20^T^ was equidistant, phylogenetically, from other members within the family *Jonesiaceae* (Fig. 2) and shared 93.0–94.8% 16S rRNA sequence similarity (Table S2). Generally, 16S rRNA gene similarity values <94.5% are recommended for the delineation of genera based on 16S rRNA gene sequence similarity [57]. Values for 16S rRNA gene, ANI and dDDH similarity below 98.8%, 95–96% and 70%, respectively, are widely accepted as thresholds for defining a prokaryotic species [36, 38, 57]. Additionally, 16S rRNA gene, AAI and PCOP values greater than 94.5%, 70.0% and 50.0%, respectively [40, 46, 48], have been proposed as thresholds for defining a prokaryotic genus. BS-2^T^ and BS-20^T^ meet the criteria for novel species; while a 16S rRNA gene similarity value of 94.8% was shared between BS-20^T^ and *T. senegalensis*, the generated phylogenetic tree (Fig. 2) and equidistant 16S rRNA gene similarity between BS-20^T^ and other genera within the broader *Jonesiaceae* continue to support the proposal that BS-20^T^ represents a novel genus. Furthermore, BS-20^T^ meets additional criteria for placement in a novel genus and species. Although AAI and POCP were introduced for genus delineation, these approaches have been criticized [51] and should not be used as the sole basis for genus delineation. These metrics should be used alongside a broader body of evidence to justify the creation of a new genus. The generated 16S rRNA gene and phylogenomic trees (Figs 2 and 4) represent a novel genus within *Jonesiaceae*.

Furthermore, across global ecological datasets, signatures of the novel organisms described here are found in <0.1% of total datasets, suggesting that bat guano is an important and understudied source for isolating novel taxa. Based on polyphasic taxonomic criteria, BS-2^T^ represents a novel species within *Lacrimispora*.

In summary, in addition to the phylogenetic analyses (Figs 1 and 3), genomic, phenotypic and predicted chemotaxonomic traits demonstrate the separation of strain BS-2^T^ from its close relatives (Tables 1, S1 and S3). It is proposed that the novel strain BS-2^T^ be named *Lacrimispora cavernae* sp. nov. Based on polyphasic taxonomic criteria, BS-20^T^ represents a novel genus within *Jonesiaceae*. In addition to the phylogenetic analyses (Figs 2 and 4), genomic, phenotypic, and chemotaxonomic traits indicate that strain BS-20^T^ is distinct from other genera within *Jonesiaceae* (Tables 2, S2 and S4). The novel strain BS-20^T^ is proposed to be named *Aleya cavernae* sp. nov.

### Description of *Lacrimispora cavernae* sp. nov.

*Lacrimispora cavernae* (ca.ver′nae. L. gen. n. *cavernae*, of a cave, referring to the habitat from which the organism was first isolated).

The cells are Gram-stain-positive, strictly anaerobic rods that are motile and spores are observed. Optimal growth is observed at 37 °C, with 0.5% (w/v) NaCl, at pH 7.0. Cells grow between 20 and 45 °C. Cells grow with up to 3.0% (w/v) NaCl. Cells grow between pH 6.0 and 8.0. The API rapid ID32A test system detected positive reactions for indole, raffinose, mannose, *N*-acetyl-*β*-glucosaminidase, *β*-glucuronidase, *α*-arabinosidase, *β*-glucosidase, *α*-glucosidase, *β*-galactosidase and *β*-galactosidase phosphatase. Negative reactions were detected for serine arylamidase, glutamyl glutamic acid arylamidase, histidine arylamidase, α-fucosidase, glutamic acid decarboxylase, glycine arylamidase, alanine arylamidase, tyrosine arylamidase, pyroglutamic acid arylamidase, leucine arylamidase, phenylalanine arylamidase, leucyl glycine arylamidase, proline arylamidase, arginine arylamidase, alkaline phosphatase, indole, nitrate reduction, raffinose, mannose, *N*-acetyl-*β*-glucosaminidase, *β*-glucuronidase, *α*-arabinosidase, β-glucosidase, *α*-glucosidase, *β*-6-gal-phosphatase, *β*-galactosidase, *α*-galactosidase, arginine dihydrolase and urease. The only major fatty acid is C_16:0_. *In silico* analysis predicts that the polar lipids are PG and DPG and the diagnostic diamino acid of the peptidoglycan is lysine.

The type strain is BS-2^T^ (=NRRL B 65694^T^=CCUG 77247^T^=CCM 9410^T^), which was isolated from a core sample of a guano pile inside Tumbling Creek Cave in Protem, MO, USA (36.6° N 92.8° W). The G+C content of the isolate was 44.1 mol%. The 16S rRNA gene and genome sequences of strain BS-2^T^ have been deposited in GenBank/EMBL/DDBJ under accession numbers PP809807.1 and GCA_040207125.1, respectively.

### Description of *Aleya* gen. nov.

*Aleya* (A.ley’a N.L. fem. N.L. fem. n. *Aleya*, named to honour Tom Aley, the name of the Tumbling Creek Cave landowner in Protem, MO, USA, for his decades-long dedication to facilitating and conducting research on natural caves and karst ecosystems).

The cells are Gram-stain positive, rod-shaped, motile, non-spore-forming and facultatively anaerobic. The major cellular fatty acids are anteiso-C_15 : 0_ and C_14:0_. *In silico* analysis predicts that the polar lipids are PG and DPG and the diagnostic diamino acid of the peptidoglycan is lysine. A member of the family *Jonesiaceae*. The type species is *Aleya cavernae*.

### Description of *Aleya cavernae* sp. nov.

*Aleya cavernae* (ca.ver′nae. L. gen. n. *cavernae*, of a cave; referring to the organism’s habitat).

In addition to the genus-level characteristics, optimal growth was observed at 25 °C with 0.5% (w/v) NaCl at pH 7.0. Cells grow between 4 and 30 °C. Cells grow with up to 4.0% (w/v) NaCl. Cells grow between pH 6.0 and 8.0. Using the API-ZYM test system, positive reactions were detected for esterase (C4), esterase lipase (C8), *N*-acetyl-*β*-glucosaminidase, *α*-mannosidase and naphthol-AS-BI-phosphohydrolase. Negative reactions were detected for alkaline phosphatase, acid phosphatase, lipase (C14), leucine arylamidase, valine arylamidase, cystine arylamidase, trypsin, *α*-chymotrypsin, *α*-galactosidase, *β*-galactosidase, *β*-glucuronidase, *α*-glucosidase, *β*-glucosidase and *α*-fucosidase.

The type strain is BS-20^T^ (=NRRL B-65699^T^=CCUG 77269^T^), which was isolated from a core sample of a guano pile inside Tumbling Creek Cave in Protem, MO, USA (36.6° N 92.8° W). The 16S rRNA gene and genome sequences of strain BS-20^T^ have been deposited in GenBank/EMBL/DDBJ under accession numbers PP337727.1 and GCA_039995105.1, respectively. The G+C content is 56.5 mol%.

## Supplementary material

10.1099/ijsem.0.007260 Supplementary Material 1.

## Abbreviations

AAI: average amino acid identity
ANI: average nucleotide identity
BHI: Brain Heart Infusion
BV-BRC: Bacterial and Viral Bioinformatics Resource Center
CCM: Czech Collection of Microorganisms
CCUG: Culture Collection of the University of Gothenburg
CDS: coding sequence
dDDH: digital DNA–DNA hybridization
DPG: diphosphatidylglycerol
KEGG: Kyoto Encyclopedia of Genes and Genomes
NRRL: Agricultural Research Service Culture Collection
OGRIs: overall genomic-based relatedness indices
PG: phosphatidylglycerol
POCP: percentage of conserved proteins
TSA: tryptic soy agar
TSB: tryptic soy broth
