# Supplementary material for: Aleya cavernae gen. nov., sp. nov., and Lacrimispora cavernae sp. nov., novel taxa isolated from bat guano
Source: Int J Syst Evol Microbiol. 2026 Aug 18;76(8):007260. doi: 10.1099/ijsem.0.007260 (PMC13488357; doi:10.1099/ijsem.0.007260)
Supplement: Supplementary file 1 [file ijsem-76-07260-s001.pdf]

**_Aleya cavernae_ gen. nov. sp. nov., and _Lacrimispora cavernae_ sp. nov., novel taxa isolated from bat guano.**


**Author Names**

Samuel Miller [1,2,f], Nam Lu[1], Payton R. Shears[1], Jacobey King [1], Krithivasan Sankaranarayanan [1,2,†], Paul A. Lawson [1*]

**Affiliations**

[1] School of Biological Sciences, 730 and 770 Van Vleet Oval, Norman, OK 73019, USA

[2] Laboratories of Molecular Anthropology and Microbiome Research, Stephenson Research and Technology Center 101 David L. Boren Blvd. Norman, OK 73019, USA

[f] Current address: Department of Microbiology and Molecular Genetics, 1110 S. Innovation Way Dr. Stillwater, OK 74074, USA

† Current Address: Wadsworth Center, NYS Department of Health, 120 New Scotland Ave. Albany, New York 12208, USA.

**Supplementary Figure 1.** Glycerophospholipid metabolism of BS-2$^{T}$ (a) and BS-20$^{T}$ (b) as determined from KEGG.

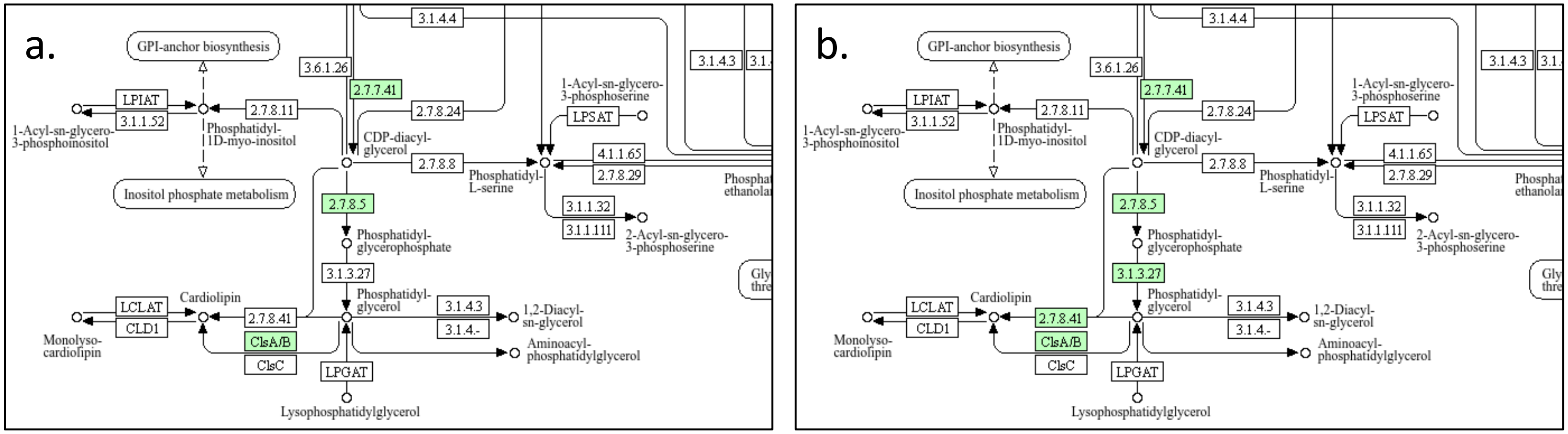

**Supplementary Table 1.** Differential genotypic characteristics of strain BS-2$^T$ and closest relatives.

Strains: 1, BS-2$^T$; 2, *Lacrimispora saccharolytica* WM1$^T$; 3, *Lacrimispora celerecrescens* DSM 5628$^T$; 4, *Lacrimispora indolis* DSM 755$^T$; 5, *Lacrimispora sphenoides* JCM 1415$^T$

| Characteristic | 1 | 2 | 3 | 4 | 5 |
|---|---|---|---|---|---|
| **16S rRNA gene Accession** | PP809807 | CP002109 | X71848 | AZUI01000001 | BBDQ01000064 |
| **16S rRNA gene simiariry (%)** | 100.0 | 98.6 | 98.5 | 98.4 | 98.3 |
| **Genome Accession** | GCA_040207125.1 | GCA_000144625.1 | GCA_002797975.1 | GCA_000526995.1 | GCA_900461315.1 |
| **dDDH (%)** | 100.0 | 29.8 | 36.5 | 31.8 | 36.6 |
| **ANI (%)** | 100.0 | 84.9 | 88.4 | 85.9 | 88.5 |
| **AAI (%)** | 100.0 | 89.0 | 88.5 | 88.2 | 91.1 |
| **G+C (%)** | 44.1 | 45.0 | 43.9 | 44.9 | 43.8 |

**Supplementary Table 2.** Differential genotypic characteristics of strain BS-20$^T$ and closest relatives.

Strains: 1, BS-20$^T$ (data from this study); *Timonella senegalensis* JC301$^T$; 3 *Sanguibacter keddieii* ST-74$^T$; 4, *Jonesia denitrificans* DSM 20603$^T$; 5, *Populibacterium corticicola* KCTC 33576$^T$; 6, *Flavimobilis marinus* JCM 12547$^T$. NR, Not reported.

| Characteristic | 1 | 2 | 3 | 4 | 5 | 6 |
|---|---|---|---|---|---|---|
| **16S Accession** | PP337727 | JN657220 | X79450 | X78420 | KT988029 | AJ783958 |
| **16S rRNA gene similarity (%)** | 100.0 | 94.8 | 93.3 | 93.1 | 93.0 | 93.3 |
| **Genome Accession** | GCA_040207125.1 | GCA_000312125.1 | GCA_000024925.1 | GCA_900475835.1 | NR | GCA_900113055.1 |
| **dDDH (%)** | 100.0 | 21.6 | 22.5 | 20.8 | NR | 22.3 |
| **ANI (%)** | 100.0 | 71.1 | 70.6 | 69.8 | NR | 69.6 |
| **AAI (%)** | 100.0 | 67.8 | 62.1 | 65.8 | NR | 61.7 |
| **POCP (%)** | 100.0 | 35.9 | 40.7 | 38.5 | NR | 41.9 |
| **G+C (%)** | 56.6 | 61.4 | 71.9 | 58.4 | 58.5 | 72.0 |

**Supplemental Table 3.** Fatty acid profile of strain BS-2$^T$ close relatives. Only fatty acids representing ≥1% of the total fatty acid content are shown. Major components (≥10%) are indicated with **bold text**.

Strains: BS-2$^T$ (this study); 2. *L.indolis* CCUG 55582$^T$ (data from CCUG); 3. *L. celerecrescens* CCUG 61015 (data from CCUG); 4. L. brassicae YZC6$^T$ (Kobayashi et al., 2024); 5. *L. aerotolerans* JCM 15733$^T$ [65]; 6. *L. algidixylanolytica* DSM 12273$^T$ [65]; 7. *L. xylanolytica* JCM 15735$^T$ [65].

| **Fatty Acid** | **1** | **2** | **3** | **4** | **5** | **6** | **7** |
|---|---|---|---|---|---|---|---|
| $C_{14:0}$ | 7.2 | 6.9 | 7.0 | 4.4 | 5.6 | 4.5 | 5.7 |
| $C_{14:0\ DMA}$ | | | | | | | 1.1 |
| $C_{15:0}$ | - | - | 1.3 | - | - | - | - |
| $C_{15:1}$ ω8*c* | 2.8 | - | - | - | - | - | - |
| $C_{15:1}$ ω6*c* | 2.2 | - | - | - | - | - | - |
| $C_{16:0}$ | **42.4** | **28.8** | **32.5** | **31.1** | **37.7** | **31.7** | **39.3** |
| $C_{16:0}$ ALDE | - | - | - | - | - | 1.4 | 1.2 |
| $C_{16:1}$ ω7*c* DMA | - | 8.9 | 6.4 | 1.8 | 2.2 | 1.9 | 1.2 |
| $C_{16:1}$ ω9*c* | 1.7 | 2.9 | 2.1 | 7.5 | 4.4 | 5.5 | 3.3 |
| $C_{16:1}$ ω9*c* DMA | - | - | - | 6.8 | **15.5** | 7.1 | **10.2** |
| $C_{16:0}$ DMA | - | - | - | 1.8 | 3.7 | 8.6 | 6.8 |
| $C_{17:0}$ anteiso | 1.3 | - | - | | | | |
| $C_{17:1}$ ω8*c* | - | 2.8 | 1.5 | - | - | - | - |
| $C_{17:1}$ ω7*c* | 4.2 | - | - | - | - | - | - |
| $C_{17:0}$ | 1.6 | - | - | - | - | - | - |
| $C_{17:1}$ ω5*c* ISO | 2.3 | 2.2 | 2.7 | - | - | - | - |
| $C_{17:0}$ 2OH | 2.0 | - | - | - | - | - | - |
| $C_{18:1}$ ω9*c* | - | - | - | 2.1 | - | 3.3 | 1.1 |
| $C_{18:1}$ ω9*c* DMA | - | - | - | 8.8 | 5.2 | 6.5 | 5.6 |
| C18:1 w7c DMA | - | **13.5** | - | - | - | - | - |
| C18:1 w11c DMA | - | - | - | **22.2** | **13.6** | **14.6** | **14.1** |
| C19:1 ISO I | - | - | 8.3 | - | - | - | - |
| Unknown 18.256 | 1.4 | - | - | - | - | - | - |
| Unknown 16.315 | 3.9 | - | - | - | - | - | - |
| Summed Feature 3* | 9.7 | 9.7 | 7.0 | - | - | - | - |
| Summed Feature 4* | 3.9 | - | - | 1.8 | 4.5 | 2.1 | 2.7 |
| Summed Feature 6* | - | - | - | - | 2.1 | - | 1.6 |
| Summed Feature 7* | | | | 1.3 | - | | |
| Summed Feature 8* | 6.9 | 6.6 | 7.2 | 3.6 | 2.2 | 2.3 | 2.4 |
| Summed Feature 10* | 2.9 | - | - | 6.8 | 4.3 | **10.5** | 2.9 |

–, Not detected or <1%. *Summed features are fatty acids that cannot be resolved reliably from other fatty acids using the chromatographic conditions chosen. The midi system groups these fatty acids together as one feature with a single percentage of the total. Summed feature 3, $C_{16:1}$ ω7c/$C_{16:1}$ ω6c; summed feature 4, unknown $C_{15.040}$/$C_{15.0}$; summed feature 6, $C_{15:0}$ anteiso 3OH/ $C_{16:1}$ ω7*c* DMA; summed feature 8, $C_{18:1}$ ω9c; summed feature 10, $C_{18:0}$/$C_{17:0}$ cyclo.

**Supplemental Table 4.** Fatty acid profile of strain BS-20$^T$ and type species of other genera within *Jonesiaceae*. Only fatty acids representing ≥1% of the total fatty acid content are shown. Major components (≥10%) are indicated with **bold text**.

Strains: 1, BS-20$^T$ (data from this study); 2 *Sanguibacter keddieii* ST-74$^T$ [53] ; 3, *Jonesia denitrificans* DSM 20603$^T$ [63]; 4, *Populibacterium corticicola* KCTC 33576$^T$ [52]; 5, *Flavimobilis marinus* JCM 12547$^T$ [64].

| **Fatty Acid** | **1** | **2** | **3** | **4** | **5** |
|---|---|---|---|---|---|
| $C_{12:0}$ | 1.2 | - | - | - | - |
| $C_{13:0}$ anteiso | - | - | 1.2 | - | 2.3 |
| iso-$C_{14:0}$ | 1.8 | 1.1 | 1.7 | - | 1.3 |
| $C_{14:0}$ | **11.5** | 5.8 | 6.7 | - | 3.9 |
| $C_{15:0}$ iso | - | 3.0 | 1.6 | - | 1.0 |
| $C_{15:0}$ | - | - | - | - | 9.3 |
| $C_{15:1}$ A anteiso | 1.9 | - | - | - | **17.7** |
| $C_{15:0}$ anteiso | **50.2** | **11.4** | **59.7** | **63.5** | **46.7** |
| $C_{16:0}$ iso | 1.8 | 5.4 | 4.3 | - | 3.9 |
| $C_{16:0}$ | 8.5 | **53.3** | **17.3** | **10.5** | **10.7** |
| $C_{17:0}$ | - | - | - | - | 1.0 |
| $C_{17:0}$ anteiso | 3.0 | 2.4 | 5.5 | - | 2.4 |
| $C_{18:0}$ | - | **10.1** | - | - | - |
| $C_{18:1}$ | - | 1.1 | - | - | - |
| $C_{20:0}$ | - | 2.2 | - | - | - |
| Summed Feature 4* | 4.7 | - | - | - | - |
| Summed Feature 5* | 1.0 | - | - | - | - |
| Summed Feature 8* | 6.1 | - | - | - | - |
| Summed Feature 10* | 1.9 | - | - | - | - |

–, Not detected or <1%. *Summed features are fatty acids that cannot be resolved reliably from other fatty acids using the chromatographic conditions chosen. The midi system groups these fatty acids together as one feature with a single percentage of the total. Summed feature 4, unknown $C_{15.040}$/$C_{15.0}$; summed feature 5, $C_{18:2}$ ω6,9c/$C_{18:0}$ ante; summed feature 8, $C_{18:1}$ ω9c, summed feature 10, $C_{18:0}$/$C_{17:0}$ cyclo.
